# MicroRNA-214 and MicroRNA-126 Are Potential Biomarkers for Malignant Endothelial Proliferative Diseases

**DOI:** 10.3390/ijms161025377

**Published:** 2015-10-23

**Authors:** Kazuki Heishima, Takashi Mori, Yukie Ichikawa, Hiroki Sakai, Yuki Kuranaga, Takayuki Nakagawa, Yuiko Tanaka, Yasuhiko Okamura, Mikio Masuzawa, Nobuhiko Sugito, Mami Murakami, Nami Yamada, Yukihiro Akao, Kohji Maruo

**Affiliations:** 1 Department of Veterinary Clinical Oncology, Faculty of Applied Biological Sciences, Gifu University, Gifu 501-1193, Japan; E-Mails: kheishima@live.jp (K.H.); lilymusashisoleil@yahoo.co.jp (Y.I.); muramami@gifu-u.ac.jp (M.M.); kmaruo@gifu-u.ac.jp (K.M.); 2 United Graduate School of Veterinary Sciences, Gifu University, Gifu 501-1193, Japan; 3 Laboratory of Veterinary Pathology, Department of Veterinary Medicine, Faculty of Applied Biological Sciences, Gifu University, Gifu 501-1193, Japan; E-Mail: shiroki@gifu-u.ac.jp; 4 United Graduate School of Drug Discovery and Medical Information Sciences, Gifu 501-1193, Japan; E-Mails: t3125010@edu.gifu-u.ac.jp (Y.K.); t3125016@edu.gifu-u.ac.jp (N.S.); namiyamada80@gmail.com (N.Y.); yakao@gifu-u.ac.jp (Y.A.); 5 Laboratory of Veterinary Surgery, Graduate School of Agricultural and Life Sciences, The University of Tokyo, Bunkyo-ku, Tokyo 113-8657, Japan; E-Mails: anakaga@mail.ecc.u-tokyo.ac.jp (T.N.); yui.tanaka.810@gmail.com (Y.T.); 6 Cooperative Department of Veterinary Medicine, Faculty of Agriculture, Iwate University, Morioka, Iwate 020-8550, Japan; E-Mail: okamura@iwate-u.ac.jp; 7 Department of Molecular Diagnostics, School of Allied Health Sciences, Kitasato University, Sagamihara, Kanagawa 252-0373, Japan; E-Mail: mikio@med.kitasato-u.ac.jp

**Keywords:** angiosarcoma, hemangiosarcoma, biomarker, microRNA, endothelial cell

## Abstract

Malignant endothelial proliferative diseases including human angiosarcoma (AS) and canine hemangiosarcoma (HSA) are serious diseases with a grave prognosis. Establishing liquid biopsy-based biomarkers for screening has definite clinical utility; however, plasma miRNAs up- or down-regulated in these sarcomas have been unclear. For identifying possible diagnostic plasma miRNAs for these sarcomas, we investigated whether plasma miR-214 and miR-126, which miRNAs play important roles in angiogenesis and tumorigenesis, were elevated in malignant endothelial proliferative diseases. For this investigation, human angiosarcoma and canine hemangiosarcoma cell lines and clinical plasma samples of canine hemangiosarcoma were examined by performing miRNA qRT-PCR. We report here that human angiosarcoma and canine hemangiosarcoma cell lines over-secreted miR-214 and miR-126 via microvesicles; in addition, their levels in the plasma samples from canines with hemangiosarcoma were increased. Moreover, the surgical resection of primary tumors decreased the levels of plasma miR-214 and miR-126. Our findings suggest that these malignant endothelial proliferative diseases over-secreted miR-214 and miR-126, thus suggesting that these miRNAs have potential as diagnostic biomarkers for malignant endothelial proliferative diseases in canine and possible in human angiosarcoma.

## 1. Introduction

Malignant endothelial proliferative diseases such as human angiosarcoma (AS) and canine hemangiosarcoma (HSA) are both serious diseases. AS shows grave prognosis with the median survival being only seven months because of the frequent local recurrence and distant metastasis [1]; however, AS has not been well studied because of its rare incidence, accounting for just 1.8% of soft tissue sarcomas [2]. The lack of case numbers makes it difficult to develop new strategies for conquering AS. HSA is a malignant endothelial proliferative disease in canines; and the one-year survival rate is less than 10% with surgery and the subsequent doxorubicin-based chemotherapy [3]. Furthermore, HSA shares many features with AS in terms of malignant behavior, HSA is a relatively common tumor unlike the low incidence of AS; therefore, HSA is a good spontaneous model for AS.

AS and HSA also share same problems regarding their diagnosis. AS, which mostly occurs in the dermis, forms well-recognizable hemorrhagic masses; however, because the clinical presentation varies widely and AS often appears to be very similar to benign hematoma or bruise-like macules, diagnosis is delayed in most patients. HSA mostly arises in visceral organs such as the spleen, where abnormality wouldn't normally be noticed, and HSA is usually diagnosed after it presents urgent clinical signs such as collapse from tumor rupture or massive hemorrhage, which results in sudden death in some cases [3]. Moreover, clinical diagnostic modalities for HSA used presently, which are cytology or diagnostic imaging techniques including ultrasound, X-ray, CT, and MRI, are unable to distinguish these malignant neoplasms from non-neoplastic lesions with certain accuracy [4]. Therefore, establishing a screening test for AS and HSA would be very useful clinically because treatment of these tumors at the early phase improves the survival rate.

MicroRNAs (miRNAs) are small non-coding RNAs, which are master-regulators of diverse biological processes including angiogenesis and tumorigenesis. Recent studies revealed that several cancers secrete microvesicles (MVs) containing specific miRNAs into the blood [5]. These miRNAs carried in MVs are protected from RNase and thus are stable in the bloodstream. Moreover, their secretion profiles in plasma reflect biological changes such as tumorigenesis, progression, and metastasis [5]; therefore, plasma miRNAs have potential to be stable, accurate, and non-invasive blood-based biomarkers for screening for malignant neoplasms. miR-214 and miR-126 are not only miRNAs playing important roles in angiogenesis and tumorigenesis intracellularly but also are released into the surrounding biofluid such as blood and serve certain functions [6,7]; therefore, these two miRNAs are candidates to be up-regulated in the plasma of malignant endothelial proliferative disease such as HSA and AS.

In this study, we demonstrated that AS and HSA cell lines over-secreted miR-214 and miR-126 via MVs. Furthermore, plasma samples obtained from the canines with HSA showed high levels of miR-214 and miR-126, suggesting that these miRNAs have potential to be biomarkers for HSA and might be applicable for diagnosing AS.

## 2. Results

### 2.1. AS and HSA Cell Lines Over-Secreted miR-214 and miR-126 via MVs

**Figure 1:**
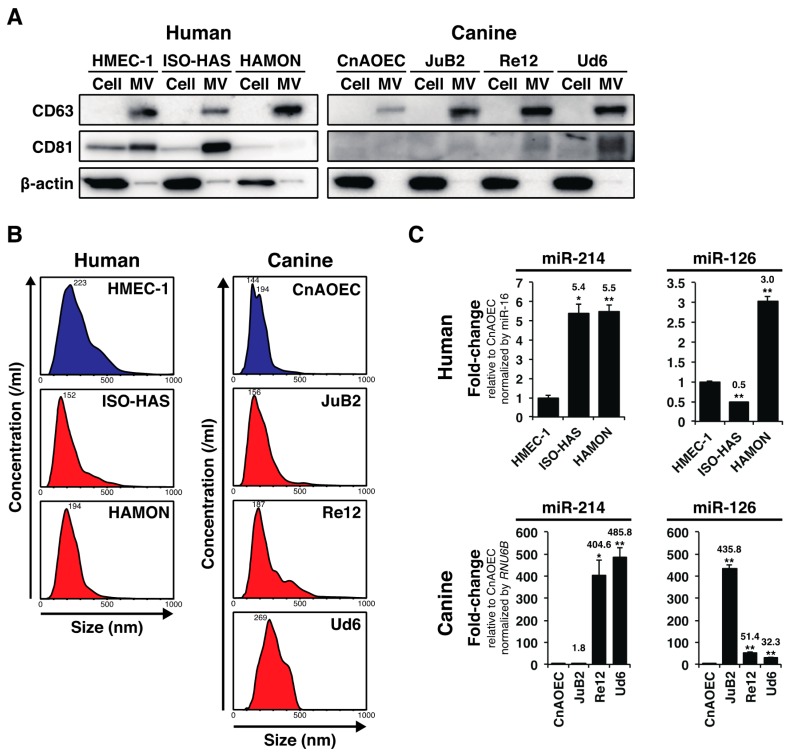
AS and HSA cell lines over-secreted miR-214 and miR-126 via MVs. (**A**) Immunoblotting for MV-markers: CD63 and CD81. The nanoparticles from all cell lines expressed CD63 or CD81 although CD81 was absent or weakly detected in MVs derived from HAMON, CnAOEC, JuB2, and Re12. β-actin was used as a negative control for excluding contamination by cellular contents; (**B**) Nanoparticle tracking analysis for AS and HSA cell lines and control ECs. The peaks of MV diameters showed a range of 144–269 nm, indicating that AS, HSA cell lines and control ECs mostly secreted MVs having a diameter of over 100 nm; (**C**) Quantitative measurement of miR-214 and miR-126 in the MVs from the conditioned media from AS, HSA cell lines and control ECs. The levels of miR-214 were significantly increased in the conditioned media of AS and HSA cell lines compared with those for the control cell lines. The levels of miR-126 were also increased likewise, except in the case of ISO-HAS. The degree of increase in canine cell lines was dramatically higher than that in human cell lines. All data are presented as the mean of triplicate experiments with error bars indicating the s.e.m. (Unpaired two-tailed *t*-test; * *p* < 0.05, ** *p* < 0.01 for comparisons with the levels of control cell lines).

miRNAs in MVs secreted by cancer cells are promising blood-based biomarkers for various tumors [5]; however, despite their clinical utility, diagnostic miRNAs up- or down-regulated in AS and HSA and thus of diagnostic importance is still unknown. AS and HSA represent the uncontrolled proliferation of neoplastic endothelial cells (ECs), and both miR-214 and miR-126 play important roles in regulating angiogenesis both intracellularly [8,9] and extracellularly [6,7]; therefore, we hypothesized that the levels of these angiogenic miRNAs are altered in the bloodstream of AS and HSA. We firstly examined whether AS (ISO-HAS [10] and HAMON [11]), HSA cell lines (JuB2, Re12, and Ud6 [12]) and control cell lines (HMEC-1 [13] and CnAOEC [12]) secreted MVs into their surrounding environment by assessing their conditioned media. We collected nanoparticles with a diameter of less than 0.45 nm from the conditioned media by filteration and ultracentrifugation and examined the levels of MV-markers including CD63 and CD81 in them by immunoblotting. As we expected, the nanoparticles from the conditioned media of all cell lines expressed CD63 or CD81 although CD81 was absent or weakly detected in MVs derived from HAMON, CnAOEC, JuB2, and Re12 (Figure 1A). The results indicate that these cell lines secrete MVs into their surrounding environment. Next, we examined the detailed characteristics of MVs by nanoparticle tracking analysis. As a result, we found that the diameter of MVs secreted from AS, HSA, and control cell lines had peaks of 144–269 nm, suggesting that these endothelial-derived cell lines mostly secreted MVs having a diameter of over 100 nm (Figure 1B). Next, by performing miRNA qRT-PCR, we assessed whether the levels of miR-214 and miR-126 in MVs were elevated in AS and HSA cell lines. As we expected, the levels of miR-214 and miR-126 were significantly increased in the media conditioned by AS and HSA cell lines compared with their levels in the control cell lines although the degree of increase in canine cell lines was dramatically higher than that in human cell lines (Figure 1C). These results suggest that both AS and HSA cells over-secreted miR-214 and miR-126 into their surrounding environment via MVs.

### 2.2. Plasma miR-16 Was a Suitable Internal Control in this Study

**Figure 2:**
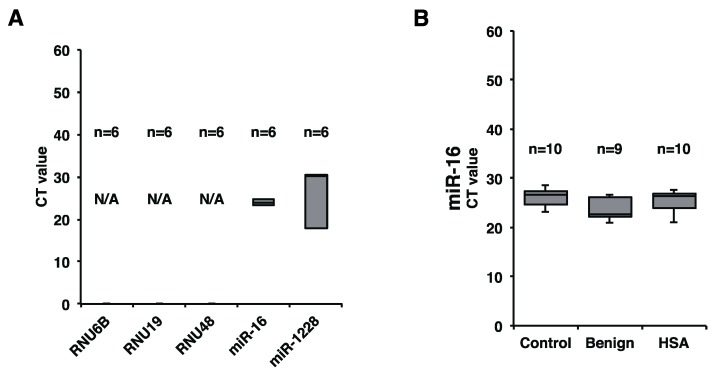
miR-16 was a suitable internal control for canine plasma miRNA analysis in this study. (**A**) Levels of *RNU6B*, *RNU19*, *RNU48*, miR-16, and miR-1228 in 6 cases (HSA, *n* = 2; Benign, *n* = 2; Control, *n* = 2), as determined by miRNA qRT-PCR. miR-16 was the most stable miRNA; (**B**) miR-16 expression in all samples used. miR-16 was stably detected in all samples. There were no significant differences among HSA, benign, and control groups (Steel-Dwass test).

We next addressed the levels of miR-214 and miR-126 in clinical plasma samples. The levels of miR-214 and miR-126 in both AS and HSA should have been examined; however, because AS is extremely rare human sarcoma, we couldn’t collect sufficient number of AS samples. Therefore, we concentrated to assess the levels of miR-214 and miR-126 in HSA, which is a spontaneous model for AS. To extract miRNAs in plasma, we used NucleoSpin^®^ miRNA Plasma (MACHEREY-NAGEL, Düren, Deutschland), which can collect both MV-miRNAs and soluble miRNAs. For assessing precise miRNA expression in the plasma, we firstly determined a suitable internal control for canine plasma miRNA. *RNU6B* is used for normalizing the level of extracellular miRNA *in vitro*; however, it is possibly unsuitable for normalizing miRNA expression in clinical cases. Therefore, we assessed the levels of candidate miRNAs for the internal control such as *RNU6B*, *RNU19*, *RNU48*, miR-16, and miR-1228 based on previous human studies [14] by using a small number of clinical samples (HSA, *n* = 2; Benign, *n* = 2; Control, *n* = 2). As a result, we judged that miR-16 was the most stable and suitable internal control miRNA among these candidates (Figure 2A). Subsequently, we assessed whether miR-16 was stably expressed in all cases used in this study. The result showed that miR-16 was also stably expressed in all samples with only a small deviation in this study (Figure 2B). Taken together, miR-16 was identified as a suitable internal control for canine plasma miRNA in this study although we have to evaluate more cases to determine that plasma miR-16 serves as a definitive internal control for canine study.

### 2.3. Plasma miR-214 and miR-126 Levels Were Significantly Increased in the Plasma of Canines with HSA

**Figure 3:**
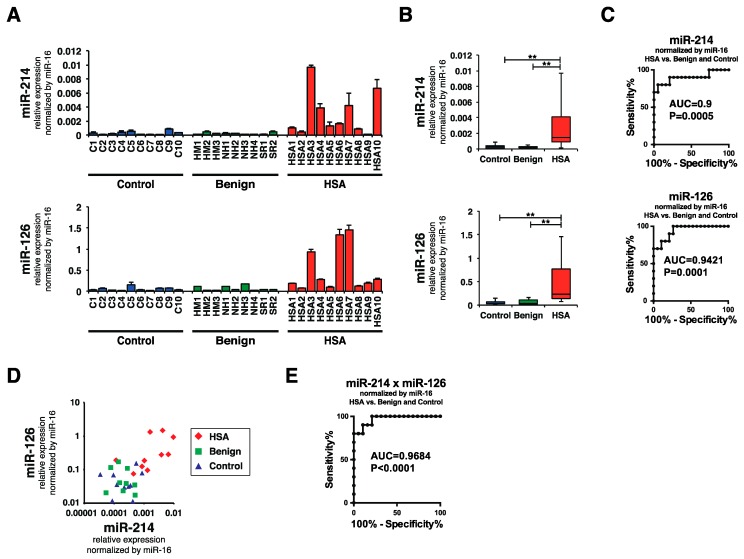
miR-214 and miR-126 was significantly increased in the plasma of HSA group. (**A**) Levels of miR-214 and miR-126 in the plasma of HSA, benign, and control groups. The levels of both miR-214 and miR-126 were significantly increased in the plasma from the HSA group compared with those for the benign and control groups; (**B**) Boxplots of the miR-214 and miR-126 levels in the plasma of HSA, benign, and control groups. The HSA group showed significantly increased levels of miR-214 and miR-126 (Steel-Dwass test; ** *p* < 0.01 for each comparison); (**C**) ROC curve analysis for the single use of miR-214 and miR-126. The AUC values of miR-214 and miR-126 were 0.9 and 0.9421, indicating the sensitivities and specificities were significantly high; (**D**) miR-214 and miR-126 showed similar profile patterns in the HSA group, which showed increased levels of both miR-214 and miR-126; (**E**) ROC curve analysis for the combination of miR-214 and miR-126. The AUC value was 0.9684, suggesting that the combination of miR-214 and miR-126 showed better accuracy than the single use of each miRNA.

**Figure 4:**
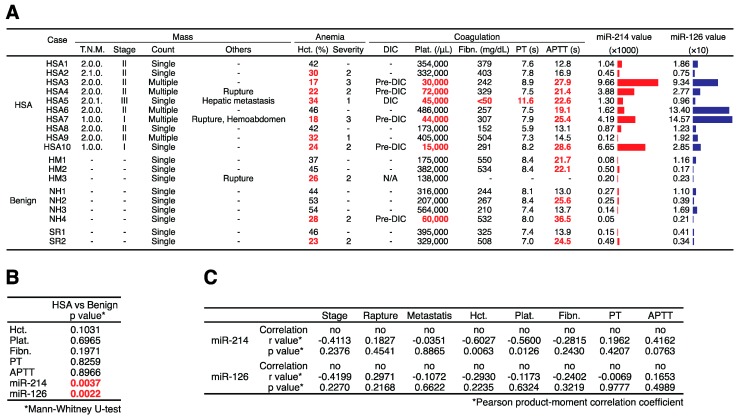
Clinical information about mass, anemia, and coagulopathy. (**A**) The clinical information of each case including the TNM classification (T.N.M.), stage, the count of mass, rupture, metastasis, hematocrit (Hct.), the severity of anemia, the state of disseminated intravascular coagulation (DIC), platelet (Plat.), fibrinogen (Fibn.), prothrombin time (PT), and activated partial thromboplastin time (APTT). The values of Hct. lower than 35% were considered as anemia and marked as red. The severity of anemia was determined based on the following criteria (Severity 1 = 30% to 35% of Hct., Severity 2 = 18% to 29% of Hct., Severity 3 = lower than 18% of Hct.). The values of Plat. lower than 100,000/μL were considered as thrombocytopenia and marked as red. The values of Fibn. lower than 150 mg/dL were considered as hypofibrinogenemia and marked as red. The values of PT longer than 10 s. and the values of APTT longer than 19 s. were considered as prolongation and marked as red. The cases were diagnosed as DIC (Pre-DIC) on the presence of together with three (two) of the following anomalies: thrombocytopenia, PT and/or APTT prolongation, and hypofibrinogenemia—considered as pre-DIC when two criteria; (**B**) The p-values regarding the differences of Hct., Plat., Fibn., PT, APTT, miR-214, and miR-126 between HSA and benign groups. There was no significant difference between HSA and benign group in the clinical conditions but in the levels of miR-214 and miR-126 (Mann-Whitney *U*-test); (**C**) The result of Pearson product-moment correlation coefficient. There was not any correlation between the levels of miRNAs and these clinical conditions.

To clarify whether the levels of miR-214 and miR-126 were increased in the plasma of canines with HSA, we examined the levels of miR-214, miR-126, and miR-16 in the plasma of canines with HSA (HSA group, *n* = 10), benign splenomegaly (benign group, *n* = 9), and in that of control canines with no specific disease (control group, *n* = 10) by performing miRNA qRT-PCR. Consistent with our hypothesis, the levels of miR-214 and miR-126 were significantly higher in the plasma of the canines with HSA than in that of the benign or control groups (Figure 3A,B). Receiver operatorating characteristic (ROC) curve analysis revealed that the area under the curve (AUC) values of miR-214 and miR-126 for discriminating HSA from benign and control groups were 0.9 and 0.9421, respectively, indicating that the sensitivities and specificities were significantly high (Figure 3C). Furthermore, since the miR-214 and miR-126 levels showed similar profile patterns (Figure 3D), the combination of miR-214 and miR126 was more effective than the single use of each miR-214 or miR-126: the AUC value was 0.9684 (Figure 3E). Next, we assessed whether the state of dogs presenting various clinical conditions affects the levels of miR-214 and/or miR-126. We firstly confirmed that there was no significant difference in their distributions of ages and weights between HSA, benign, and control group (Table 1). Subsequently, we evaluated whether there was significant difference in these values between HSA and benign groups by collecting the clinical information including hematocrit (Hct.), platelet (Plat.), fibrinogen (Fibn.), prothrombin time (PT), and activated partial thromboplastin time (APTT) in HSA and benign group from their medical records. As a result, we found that there was no significant difference between HSA and benign group in the clinical conditions but in the levels of miR-214 and miR-126 (Figure 4A,B); furthermore, the result of Pearson product-moment correlation coefficient showed that there was not any correlation between the levels of miRNAs and these clinical conditions (Table 1), suggesting that the levels of these miRNAs does not correlate with the state of anemia and coagulopathy. Taken together, these results suggest that both miR-214 and miR-126 have potential to be biomarkers for discriminating HSA from benign or healthy cases.

**Table 1:** Case information of age and weight. The case information of age and weight used in this study. There was no significant difference in their ages and weights between HSA, benign, and control groups (Steel-Dwass test).

	Age (y.m.)				Weight (kg)					*p* Value *	
	**Mean**	**Median**	**s.d.**		**Mean**	**Median**	**s.d.**			**Age**	**Weight**
HSA	10.6.	10.9.	2.8.		11.1	6.6	9.6		HSA *vs.* Benign	>0.05	>0.05
Benign	10.1.	11.1.	2.6.		10.2	6.8	9.6		Benign *vs.* Control	>0.05	>0.05
Control	10.3.	10.3.	2.8.		6.9	5.4	3.3		HSA *vs.* Control	>0.05	>0.05

* Steel-Dwass test.

### 2.4. Plasma miR-214 and miR-126 Levels Were Decreased after the Surgical Resection of Primary Splenic HSA

**Figure 5:**
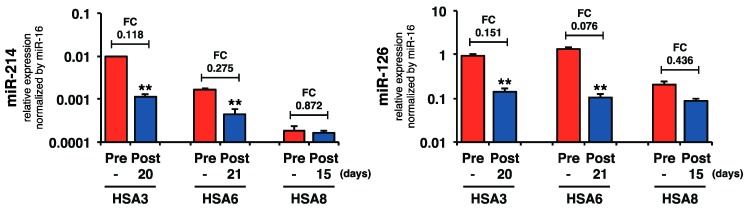
Surgical resection of the primary tumor decreased the levels of plasma miR-214 and miR-126. Surgical resection of primary tumor significantly decreased the levels of both miR-214 and miR-126 in two out of three cases shown. (Paired two-tailed *t*-test; ** *p* < 0.01 for comparisons with the levels of pre-operation samples).

Because the plasma miR-214 and miR-126 levels were increased in canines with HSA, we assessed whether the surgical resection of primary splenic HSA would affect these levels. We examined the levels of miR-214 and miR-126 in paired (pre- and post-operation) plasma samples from three cases (HSA3, HSA6, and HSA8). The results showed that the surgical resection of the primary tumor significantly decreased the levels of both miR-214 and miR-126 in two out of three cases (Figure 5), supporting our hypothesis that HSA cells secreted the miR-214 and miR-126 into the bloodstream.

Taken together, we demonstrated that AS and HSA cell lines over-secreted miR-214 and miR-126 via MVs. We identified miR-214 and miR-126 as over-secreted miRNAs in the plasma of canines with HSA, which is a spontaneous model of AS. These results suggest that both miR-214 and miR-126 have potential to be biomarkers for the diagnosis of HSA and possibly be applicable for diagnosing AS.

## 3. Discussion

Establishing a miRNA-based screening test has definite clinical significance; however, circulating miRNAs, which are up- or down-regulated in HSA and AS, has been unclear. In this study, we examined whether the miRNAs regulating angiogenesis such as miR-214 and miR-126 have potential to be biomarkers for AS and HSA. Here, we demonstrated that AS and HSA cell lines over-secreted miR-214 and miR-126 via MVs. Furthermore, we found that the levels of these miRNAs were also high in the plasma of canines with HSA, which is a spontaneous model of AS.

miR-214 and miR-126 plays important roles in regulating angiogenic pathways both intracellularly and extracellularly. Several human cancers reportedly secrete miR-214 or miR-126 into the bloodstream via MVs and/or as a soluble miRNA [15,16]. In addition, extracellular miR-214 and miR-126 work for promoting angiogenesis and suppressing senescence in ECs [7] or promoting metastasis by modulating adhesive activities in ECs [6]. Based on these reports, we hypothesized that the levels of these two miRNAs would be up-regulated and have potential to be blood-based biomarkers for endothelial proliferative diseases such as AS and HSA. Consistent with our hypothesis, we found that both HSA and AS cell lines over-secreted miR-214 and miR-126; and their plasma levels were also higher in the canines with HSA than in those of the benign and control groups, although this finding may have reflected the amount of both MV- and soluble miRNA. The result of clinical samples of HSA suggests that miR-214 and miR-126 are potential biomarkers for HSA; furthermore, given their extracellular functions, the over-secretion of miR-214 and miR-126 may function to accelerate the uncontrolled proliferation of neoplastic and tumor-associated ECs in a paracrine manner.

Unfortunately, we were not able to evaluate the levels of miR-214 and miR-126 in AS patients because of the rare incidental rate; however, judging from results of AS cell lines and clinical samples of HSA, plasma miR-214 and miR-126 might be increased in AS as well as HSA although the levels of these miRNAs should be evaluated in the plasma of AS patients. We found that the degree of up-regulation of these miRNAs was higher in HSA cell lines than that in AS cell lines; however, it is still ambiguous that this result reflects the differences of clinical nature between AS and HSA because the AS cell lines used in this study only proliferate under very limited condition and shows slower growth rate than HSA cell lines we used.

Interestingly, the levels and functions of extracellular miRNAs is not always corresponding with these of intracellular miRNAs. We previously reported that miR-214 was down-regulated in the clinical samples and cell lines of HSA [17]; however, in this study, we found that miR-214 was over-secreted in the plasma samples. Similar discrepancy was also observed in human cancers; for example, Yamada *et al.* reported that miR-1246 was down-regulated in human colorectal cancer tissues and cell lines; however, the level of miR-1246 in the plasma from colorectal cancer patients was conversely elevated [18]. Moreover, unlike the extracellular angiogenic function of miR-214, the intracellular miR-214 works as a negative regulator of angiogenesis [8], and miR-214 was reportedly an anti-oncomir inducing apoptosis by targeting COP1-p53 axis in HSA [17]. Their intracellular and extracellular functions seem to be contradictory; however, because miRNAs have many targets, their functions may change depending on the state of target cells or their co-factors including other miRNAs and proteins in MVs. Although the detailed mechanism of these paradoxical levels and functions is still unclear, these findings may indicate that there is a mechanism that specifically secretes unnecessary or harmful anti-oncomir in tumor cells to their surrounding environment for promoting angiogenesis by affecting other normal ECs.

In this study, the levels of miR-214 and miR-126 didn’t correlate with their clinical conditions such as anemia and coagulopathy; moreover, the resection of primary tumors decreased the levels of miR-214 and miR-126 in the plasma, suggesting that these levels precisely reflected the biological change (resection) of the primary tumor site but not their clinical conditions such as anemia and coagulopathy. 

Biomarkers for diagnosing endothelial proliferative diseases have been sought, although they have not been well-established yet. The levels of troponin I [19] and plasma VEGF [20] are reportedly elevated in HSA plasma; however, their sensitivities and specificities are not adequate to discriminate HSA clinically from other non-neoplastic diseases. Other protein-based approaches measuring serum thymidine kinase activity [21] or big endothelin-1 [22] are relatively accurate to discriminate malignant lesions from tumor-like lesions with 0.92 and 0.93 AUC values, respectively. In this study, we found that plasma miR-214 and miR-126 were able to discriminate the HSA from non-neoplastic lesions and healthy control groups with high accuracy, which AUC value were all over 0.9. These results suggest that plasma miR-214 and miR-126 would be potential biomarkers for diagnosing HSA although we need to evaluate more cases to establish these miRNAs as clinical biomarkers.

In conclusion, we demonstrated that AS and HSA cell lines over-secreted miR-214 and miR-126 via MVs. Moreover, the levels of miR-214 and miR-126 were markedly high in the plasma of canines with splenic HSA. Our findings suggest these 2 miRNAs to be potential diagnostic biomarkers for HSA and might be applicable for AS.

## 4. Experimental Section

### 4.1. Cell Lines Used and Normal Culture Conditions

The HSA cell lines used, JuB2, Re12 and Ud6 [12], were cultured in D-MEM with 10% fetal bovine serum (FBS; Sigma-Aldrich, St. Louis, MO, USA). Canine aortic endothelial cells (CnAOECs), used as normal canine ECs, were purchased from Cell Applications Inc. (San Diego, CA, USA) and cultured in canine endothelial cell basal medium with growth supplements. The AS cell lines used, ISO-HAS [10] and HAMON [11], and the human normal EC line HMEC-1 [13] were cultured in EBM-2 containing the MV-BulletKit (CC-3202, Lonza, Basel, Switzerland).

### 4.2. Preparation of MV-Free Culture Media

Following the centrifugation at 3000 rpm for 5 min, FBS and the growth supplements for CnAOEC and MV-BulletKit were filtered through a Millex-HV Filter with 0.45-μm pores. The residual MVs were removed by ultracentrifugation at 100,000 rpm for 3 h. Finally, the MV-free FBS or growth supplements were added to the each medium.

### 4.3. Isolation of Cell-Derived Nanoparticles 

All cell lines were seeded as 1.0 × 10^6^ cells per culture-dish and cultured in the corresponding MV-free medium for 72 h at 37 °C. After centrifugation of the conditioned media at 3000 rpm for 5 min, the supernatant was filtered through a 0.45-μm-pore filter to remove the large particles such as cell debris. The nanoparticles were then collected by ultracentrifugation at 100,000 rpm for 3 h and used for subsequent analysis.

### 4.4. Immunoblotting

The pellets containing nanoparticles were lysed in RIPA-buffer containing Protease Inhibitor Cocktail (Sigma-Aldrich) and Phosphatase Inhibitor Cocktail (nakarai tesque, Kyoto, Japan). The concentrations of protein were quantified by performing the Bradford protein assay (DC Protein assay kit, Bio-Rad, Hercules, CA, USA). Protein samples (5 μg/lane) were loaded into 10%–15% sodium dodecylsulphate-polyacrylamide gels. Subsequently, the samples were separated by electrophoresis (SDS-PAGE) and then transferred to 0.45-µm polyvinylidene fluoride membranes (Immobilon-P Membrane, EMD Millipore, Billerica, MA, USA). The membranes were placed in 5% non-fat dry milk diluted by phosphate buffered saline (PBS) containing 0.1% Tween-20 (TBS-T) for 1 h to block the non-specific reactions. The membranes were incubated overnight at 4 °C with the desired primary antibody afterword. The primary antibodies used for immunoblotting were anti-CD63 mouse-monoclonal antibody (clone RFAC4, Cat.#CBL553, EMD Millipore), anti-CD83 mouse-monoclonal antibody (clone B-11, Cat.#sc-166029, Santa Cruz, CA, USA), and anti-β-actin mouse-monoclonal antibody (clone AC-74, Cat.#A5316, Sigma-Aldrich). After washing the membranes to remove unbound antibody by TBST, the membranes were exposed to secondary horseradish peroxidase-linked anti-mouse IgG antibodies (Cell Signaling Technology, Danvers, MA, USA) diluted 1:1000 for 1 h at room temperature. After washing the membranes three times with TBS-T, the proteins on the membrane were visualized by using Luminata Forte Western HRP substrate (EMD Millipore).

### 4.5. Nanoparticle Tracking Analysis

The pellets containing nanoparticles were suspended in 500 μL of PBS and then diluted at 1:1000 in PBS. The samples were analyzed by using NanoSight (NTA Version 2.3, Wiltshire, UK) according to the provided instructions.

### 4.6. RNA Extraction from the Conditioned Media 

The conditioned media containing cell-derived nanoparticles were filtered by using an ExoMir™ kit (Bioo Scientific Corporation, Austin, TX, USA). The total RNA from the conditioned medium was extracted from 22-nm filters according to the standard protocol.

### 4.7. Collection and Handling of Clinical Samples 

Plasma samples were obtained from canines diagnosed as HSA (*n* = 10) or benign splenomegalies (*n* = 9) including hematoma (*n* = 3), nodular hyperplasia (*n* = 4), and splenorrhagia (*n* = 2) at the Animal Medical Center of Gifu University, the Veterinary Medical Center of The University of Tokyo or Iwate University Veterinary Teaching Hospital. All canines were histopathologically diagnosed as HSA or non-neoplastic splenic diseases by at least one JCVP-board-certified pathologist. Control canines, which had no specific disease, were obtained as control blood, representing healthy dogs (*n* = 10), from a private clinic in Gifu. To avoid artificial hemolysis of samples, all blood samples were carefully taken from a jugular or cephalic vein by syringe equipped with a 21G needle. Subsequently, whole-blood samples were added to EDTA tubes and gently mixed at room temperature. The samples were then centrifuged at 3000 rpm for 20 min at 4 °C. The plasma was carefully removed to a new 1.5-mL microfuge tube, and stored at −80 °C. The state of hemolysis in collected samples was evaluated and visually recognizable hemolytic samples were excluded in this study. The clinical information including T.N.M., stage, the count of mass, rupture, metastasis, hematocrit (Hct.), the severity of anemia, the state of DIC, platelet (Plat.), fibrinogen (Fibn.), prothrombin time (PT), and activated partial thromboplastin time (APTT) was collected from their medical records. The detailed information about sex, age, weight, breed, and histological diagnosis of the each case is given in Figure S1. All clinical samples were collected from a portion of biopsies for diagnosis. The sampling and application for this study of all clinical materials were carried out with the owner’s consent based on the guidelines that have been approved by the committee of each university.

### 4.8. RNA Extraction from Plasma

Total plasma RNA was extracted by using NucleoSpin^®^ miRNA Plasma (MACHEREY-NAGEL) according to the standard protocol.

### 4.9. miRNA Quantitative Real-Time Polymerase Chain Reaction (miRNA qRT-PCR)

For determining the levels of the miRNAs, total RNA was firstly reverse-transcribed to cDNA with TaqMan^®^ MicroRNA Reverse Transcription Kit (Applied Biosystems^®^, Thermo Fisher Scientific, Waltham, MA, USA). Subsequently, the levels of miR-214 (AB Assay ID 002306), miR-126 (AB Assay ID 002228), *RNU6B* (AB Assay ID 001093), *RNU19* (AB Assay ID 001003), *RNU48* (AB Assay ID 001006), miR-16 (AB Assay ID 000391), and miR-1228 (AB Assay ID 002919) were determined with TaqMan^®^ MicroRNA Assays (Applied Biosystems^®^) and a TaKaRa Thermal Cycler Dice^®^ Real Time System I (Takara, Shiga, Japan). The 2^−Δ*C*t^ method was used for calculating the relative quantities. Each measurement was done in triplicate.

### 4.10. Statistical Analysis

For each examination, differences were evaluated by performing different statistical analyses. The unpaired two-tailed *t*-test was used in Figure 1C. The paired two-tailed *t*-test was used in Figure 5. The Steel-Dwass test was used in Figure 2B and Figure 3B, and Table 1. Mann-Whitney *U*-Test was used in Figure 4B. ROC curve analysis was used in Figure 3C,E. Pearson product-moment correlation coefficient was used in Figure 4C. The *p*-value of less than 0.05 was considered to be significant in this study.

## Supplementary Materials

Supplementary materials can be found at http://www.mdpi.com/1422-0067/16/10/25377/s1.

## References

[1] Abraham J.A., Hornicek F.J., Kaufman A.M., Harmon D.C., Springfield D.S., Raskin K.A., Mankin H.J., Kirsch D.G., Rosenberg A.E., Nielsen G.P. (2007). Treatment and outcome of 82 patients with angiosarcoma. Ann. Surg. Oncol..

[2] Coindre J.M., Terrier P., Guillou L., Le Doussal V., Collin F., Ranchere D., Sastre X., Vilain M.O., Bonichon F., N'Guyen Bui B. (2001). Predictive value of grade for metastasis development in the main histologic types of adult soft tissue sarcomas: A study of 1240 patients from the French Federation of Cancer Centers Sarcoma Group. Cancer.

[3] Hammer A.S., Couto C.G., Filppi J., Getzy D., Shank K. (1991). Efficacy and toxicity of VAC chemotherapy (vincristine, doxorubicin, and cyclophosphamide) in dogs with hemangiosarcoma. J. Vet. Intern. Med..

[4] Ivancic M., Long F., Seiler G.S. (2009). Contrast harmonic ultrasonography of splenic masses and associated liver nodules in dogs. J. Am. Vet. Med. Assoc..

[5] Heneghan H.M., Miller N., Kerin M.J. (2010). MiRNAs as biomarkers and therapeutic targets in cancer. Curr. Opin. Pharmacol..

[6] Taverna S., Amodeo V., Saieva L., Russo A., Giallombardo M., De Leo G., Alessandro R. (2014). Exosomal shuttling of miR-126 in endothelial cells modulates adhesive and migratory abilities of chronic myelogenous leukemia cells. Mol. Cancer.

[7] Van Balkom B.W., de Jong O.G., Smits M., Brummelman J., den Ouden K., de Bree P.M., van Eijndhoven M.A., Pegtel D.M., Stoorvogel W., Wurdinger T. (2013). Endothelial cells require miR-214 to secrete exosomes that suppress senescence and induce angiogenesis in human and mouse endothelial cells. Blood.

[8] Van Mil A., Grundmann S., Goumans M.J., Lei Z., Oerlemans M.I., Jaksani S., Doevendans P.A., Sluijter J.P. (2012). MicroRNA-214 inhibits angiogenesis by targeting Quaking and reducing angiogenic growth factor release. Cardiovasc. Res..

[9] Fish J.E., Santoro M.M., Morton S.U., Yu S., Yeh R.F., Wythe J.D., Ivey K.N., Bruneau B.G., Stainier D.Y., Srivastava D. (2008). miR-126 regulates angiogenic signaling and vascular integrity. Dev. Cell.

[10] Masuzawa M., Fujimura T., Hamada Y., Fujita Y., Hara H., Nishiyama S., Katsuoka K., Tamauchi H., Sakurai Y. (1999). Establishment of a human hemangiosarcoma cell line (ISO-HAS). Int. J. Cancer.

[11] Hoshina D., Abe R., Yoshioka N., Saito N., Hata H., Fujita Y., Aoyagi S., Shimizu H. (2013). Establishment of a novel experimental model of human angiosarcoma and a VEGF-targeting therapeutic experiment. J. Dermatol. Sci..

[12] Murai A., Asa S.A., Kodama A., Hirata A., Yanai T., Sakai H. (2012). Constitutive phosphorylation of the mTORC2/Akt/4E-BP1 pathway in newly derived canine hemangiosarcoma cell lines. BMC Vet. Res..

[13] Ades E.W., Candal F.J., Swerlick R.A., George V.G., Summers S., Bosse D.C., Lawley T.J. (1992). HMEC-1: Establishment of an Immortalized Human Microvascular Endothelial Cell Line. J. Investig. Dermatol..

[14] Hu J., Wang Z., Liao B.Y., Yu L., Gao X., Lu S., Wang S., Dai Z., Zhang X., Chen Q. (2014). Human miR-1228 as a stable endogenous control for the quantification of circulating microRNAs in cancer patients. Int. J. Cancer.

[15] Sharma T., Hamilton R., Mandal C.C. (2015). miR-214: A potential biomarker and therapeutic for different cancers. Future Oncol..

[16] Cortez M.A., Bueso-Ramos C., Ferdin J., Lopez-Berestein G., Sood A.K., Calin G.A. (2011). MicroRNAs in body fluids—The mix of hormones and biomarkers. Nat. Rev. Clin. Oncol..

[17] Heishima K., Mori T., Sakai H., Sugito N., Murakami M., Yamada N., Akao Y., Maruo K. (2015). MicroRNA-214 Promotes Apoptosis in Canine Hemangiosarcoma by Targeting the COP1-p53 Axis. PLoS ONE.

[18] Yamada N., Tsujimura N., Kumazaki M., Shinohara H., Taniguchi K., Nakagawa Y., Naoe T., Akao Y. (2014). Colorectal cancer cell-derived microvesicles containing microRNA-1246 promote angiogenesis by activating Smad 1/5/8 signaling elicited by PML down-regulation in endothelial cells. Biochim. Biophys. Acta.

[19] Chun R., Kellihan H.B., Henik R.A., Stepien R.L. (2010). Comparison of plasma cardiac troponin I concentrations among dogs with cardiac hemangiosarcoma, noncardiac hemangiosarcoma, other neoplasms, and pericardial effusion of nonhemangiosarcoma origin. J. Am. Vet. Med. Assoc..

[20] Clifford C.A., Hughes D., Beal M.W., Mackin A.J., Henry C.J., Shofer F.S., Sorenmo K.U. (2001). Plasma vascular endothelial growth factor concentrations in healthy dogs and dogs with hemangiosarcoma. J. Vet. Intern. Med..

[21] Thamm D.H., Kamstock D.A., Sharp C.R., Johnson S.I., Mazzaferro E., Herold L.V., Barnes S.M., Winkler K., Selting K.A. (2012). Elevated serum thymidine kinase activity in canine splenic hemangiosarcoma*. Vet. Comp. Oncol..

[22] Fukumoto S., Miyasho T., Hanazono K., Saida K., Kadosawa T., Iwano H., Uchide T. (2015). Big endothelin-1 as a tumour marker for canine haemangiosarcoma. Vet. J..

