# Supplementary material for: MicroRNA-214 and MicroRNA-126 Are Potential Biomarkers for Malignant Endothelial Proliferative Diseases
Source: Int J Mol Sci. 2015 Oct 23;16(10):25377–91. doi: 10.3390/ijms161025377 (PMC4632806; doi:10.3390/ijms161025377)
Supplement: Supplementary file 1 [file ijms-16-25377-s001.pdf]

Supplementary Information

Code	Sex	Age (y,m)	Weight (kg)	Breed	Histological diagnosis	Other findings
HSA1	CM	10y9m	23.46	Golden retriever	Splenic hemangiosarcoma	-
HSA2	M	10y8m	31.6	Golden retriever	Splenic hemangiosarcoma	-
HSA3	M	5y	3.7	Maltese	Splenic hemangiosarcoma	-
HSA4	CM	8y8m	7	Miniature dachshund	Splenic hemangiosarcoma	-
HSA5	CM	11y7m	15.7	French bulldog	Splenic hemangiosarcoma	-
HSA6	CM	11y1m	6.2	Miniature dachshund	Splenic hemangiosarcoma	-
HSA7	SF	8y6m	5	Miniature dachshund	Splenic hemangiosarcoma	-
HSA8	SF	13y4m	11.06	Welsh corgi	Splenic hemangiosarcoma	-
HSA9	SF	14y6m	5	Miniature schnauzer	Splenic hemangiosarcoma	-
HSA10	SF	10y10m	2.66	Miniature dachshund	Splenic hemangiosarcoma	-
HM1	F	13y8m	5.9	Papillon	Hematoma	-
HM2	M	15y3m	4.12	Miniature dachshund	Hematoma	-
HM3	M	7y	5.38	Mix	Hematoma	
NH1	F	11y1m	8.85	Shiba	Nodular hyperplasia	-
NH2	CM	11y3m	7.4	Jack Russell Terrier	Nodular hyperplasia	-
NH3	M	9y	15.7	Welsh corgi	Nodular hyperplasia	Extramedullary hematopiesis
NH4	SF	9y6m	3.4	Papillon	Nodular hyperplasia	-
SR1	M	9y8m	33.9	Labrador retriever	Splenorrhagia	-
SR2	M	11y9m	6.8	Shih-tzu	Splenorrhagia	Extramedullary hematopiesis
C1	SF	10y5m	12.92	Beagle	-	-
C2	CM	10y11m	5.24	Miniature dachshund	-	-
C3	M	10y	5.64	Miniature dachshund	-	-
C4	CM	9y4m	4.68	Toy poodle	-	-
C5	SF	13y	3.84	Miniature dachshund	-	-
C6	M	11y11m	9.4	Miniature schnauzer	-	-
C7	F	5y1m	4.14	Toy poodle	-	-
C8	SF	7y11m	4.9	Toy poodle	-	-
C9	M	9y8m	6.52	Miniature dachshund	-	-
C10	M	14y7m	11.65	Mix	-	-

Figure S1. Additional case information. The information of each canine case used in this study regarding their sex, age, weight, breed, and histological diagnosis.
